# The return of neuropsychiatry: why clinical neuroscience needs neurology and psychiatry reunited

**DOI:** 10.1093/brain/awag324

**Published:** 2026-11-04

**Authors:** David Belin, Masud Husain

**Affiliations:** Editor-in-Chief, Brain Communications, Cambridge, UK; Editor-in-Chief, Brain, Oxford, UK

The distinction between neurology and psychiatry is so deeply ingrained that it is sometimes assumed to represent a fundamental truth about the organization of brain diseases, their diagnosis and treatment.^1,2^ Patients with Parkinson’s disease see neurologists; patients with depression are referred to psychiatrists. One group is perceived to treat disorders of the brain, the other disorders of the mind. Medical schools maintain separate curricula, hospitals separate departments, funding agencies separate review panels, and specialist societies separate annual meetings. Insights into the semiology and pathophysiology of disorders of the CNS gained over the past 40 years warrant a reconsideration of this dichotomic approach to clinical neuroscience, not least because conditions that were once considered prototypical neurological disorders, such as Parkinson’s disease, have since been demonstrated also to encompass many psychiatric comorbid elements.^3^

Ironically, at the time *Brain* was launched as a home for transformative clinical and translational neuroscience nearly 150 years ago, physicians sought to understand the relationship between brain pathology and behaviour without regard for the boundaries that later came to define medical practice.^1^ Broca's description of expressive aphasia, Wernicke's studies of language comprehension, Hughlings Jackson's hierarchical organization of the nervous system, Charcot's analyses of hysteria and movement disorders, Meynert's neuroanatomical investigations and Kraepelin's biological classification of mental illness all emerged from the same intellectual tradition. These pioneers differed in their interests but shared a common conviction: disorders of the mind were inseparable from disorders of the brain. Even Sigmund Freud, whose work would later contribute to the separation of psychiatry from neurology, began his career as a neurologist studying the anatomy of the nervous system.^4^ His turn towards psychoanalysis reflected not a rejection of biology but an acknowledgement of its limitations.

Neurology and psychiatry emerged from the same parent discipline to address different manifestations of dysfunction within the same organ, the former dealing with the consequences of physical alterations of the central and/or peripheral nervous system, the latter with functional alterations of the brain.

Thus, neurology developed around disorders with a visible, biological basis. Stroke, epilepsy, tumours and multiple sclerosis could be localized, examined and, increasingly, explained by neuropathology, observable at post-mortem or under the microscope. Psychiatry inherited conditions the underlying pathophysiological basis of which remained invisible: schizophrenia, depression, anxiety disorders, addiction and obsessive-compulsive disorder. Faced with disorders that lacked obvious lesions or pathological signatures, in the absence of tools capable of interrogating the neural basis of brain function *in vivo*, psychiatry naturally developed explanatory models rooted in psychology rather than neurobiology. The divergence between the specialties was therefore not primarily philosophical. It was technological.

Nevertheless, separate residency and training programmes evolved for the specialties of neurology and psychiatry. Different methods of assessment and examination, vocabularies and professional identities emerged. Neurologists became experts in localization and neuropathology. Psychiatrists embraced phenomenology and behaviour. Although both disciplines treated diseases of the brain, they increasingly appeared to inhabit different intellectual worlds. For much of the 20th century, this division seemed reasonable. It no longer does.

The modern era of neuroscience, in particular translational neuroscience, propelled by increasingly sophisticated behavioural neuroscience, has fundamentally altered the way we can interrogate brain structure and function in health and disease. The resulting ever-increasing understanding of the biological basis of behaviour across different levels of integration of brain function, from neural systems, to circuits, to cells, to molecules and even genes, across cell types, provides a fertile ground for a more mechanistic understanding of disorders of the brain across the neuropsychiatric spectrum.

The wealth of knowledge about the complexity of the presentation of neurological and psychiatric conditions, which are rarely devoid of comorbid symptoms, together with genetics, has demonstrated extensive overlap in susceptibility across neurological and psychiatric diseases. Structural and functional neuroimaging have revealed reproducible abnormalities across virtually every major psychiatric disorder. Molecular neuroscience has uncovered common mechanisms involving synaptic plasticity, neuroimmune signalling and neural development, while systems neuroscience has shifted attention away from isolated anatomical regions towards distributed neural circuits and networks, the dysfunction of which can give rise to diverse clinical syndromes, such as compulsion^5^ or anhedonia^6^ across nosological boundaries.

For example, once viewed principally through the lens of movement disorders such as Parkinson’s disease or Huntington’s disease, corticostriatal circuitry is now also recognized to be a central neural component of the pathophysiology of obsessive-compulsive disorder, addiction, Tourette syndrome, and a range of impulsive and compulsive behaviours.^5^ Limbic-prefrontal networks are implicated in major depression, anxiety disorders, temporal lobe epilepsy and traumatic brain injury. Dysfunction of salience networks has been described in schizophrenia, frontotemporal dementia, addiction and autism spectrum disorders.^7^ Neural circuits appear remarkably indifferent to nosological or specialization boundaries.

At the clinical level, the same convergence has occurred: Parkinson's disease is no longer defined simply by tremor and rigidity but by depression, anxiety, psychosis, apathy, impulse-control disorders and cognitive impairment. Patients with frontotemporal dementia often present to psychiatrists years before neurological symptoms become apparent. Autoimmune encephalitis frequently masquerades as primary psychosis. Epilepsy clinics manage depression and anxiety as routinely as seizures. Conversely, cognitive impairment, altered sensory processing, sleep disturbance and measurable abnormalities of functional connectivity have become integral to our understanding of schizophrenia, mood disorders and addiction.

The historical distinction between neurological and psychiatric disease increasingly reflects where patients enter the healthcare system rather than the biology of their illness. Indeed, one could argue that both specialties have been evolving towards each other from opposite directions. Neurology has progressively expanded beyond localization. Contemporary neurological practice encompasses cognition, behaviour, mood, quality of life and social functioning. Neurologists now routinely employ biomarkers, digital technologies, sometimes even functional brain network analyses, and multidimensional outcome measures that extend far beyond classical neurological examination. Psychiatry, meanwhile, has become increasingly biological. Neuroimaging, genetics, electrophysiology, computational modelling and circuit-based neuromodulation have become integral components of psychiatric research.

This slow convergence between the two fields is perhaps epitomized best in the adoption by neurologists and psychiatrists of neuromodulation techniques, such as deep brain stimulation^8^ and repetitive transcranial magnetic stimulation,^9,10^ which unequivocally alter the function of neural systems, to treat motor symptoms in Parkinson’s disease as well as depression or compulsions, sometimes targeting similar structures, such as the subthalamic nucleus.

This does not imply that psychiatry should become neurology, nor that neurology should absorb psychiatry. Rather, it is an argument that both disciplines are converging towards a common scientific framework: one in which brain disorders are understood as different manifestations of dysfunction within overlapping, if not the same, neurobiological systems. And perhaps one returning towards its historical, unified origin.

Neurology and psychiatry have each developed invaluable rich clinical traditions. Neurological examination remains one of medicine's greatest diagnostic achievements. Psychiatry's sophisticated understanding of phenomenology, interpersonal relationships and psychological formulation has transformed the care of patients with complex behavioural disorders. Neither discipline should lose these strengths. Rather, each should recognize that they represent complementary approaches to understanding disorders of the same organ.

History reminds us that the separation of neurology and psychiatry was not inevitable. It emerged because 19th-century medicine lacked the tools to investigate the neural basis of behaviour. Today, we possess some of those tools, including sophisticated preclinical models of several neuropsychiatric disorders. Advances in multimodal neuroimaging, human genetics, single-cell transcriptomics, connectomics, computational modelling and artificial intelligence are revealing the architecture of brain disorders with unprecedented resolution. As these approaches mature, diagnostic categories themselves are likely to become increasingly dimensional, determined by multidimensional biomarkers of underlying syndromes rather than specific symptoms.

The future will belong to clinicians and scientists capable of integrating molecular biology, systems neuroscience, cognition, behaviour and lived experience into a unified understanding of brain disease. Such an approach, which is not revolutionary, in that it represents a return to the theoretical foundations of 19th century clinical neuroscience, requires a repository of this unified knowledge, and we believe it is what *Brain* and *Brain Communications* should be.

Perhaps, then, the greatest irony is that the future of clinical neuroscience lies not in creating a new discipline but in rediscovering the one from which both neurology and psychiatry originally emerged. We want *Brain* and *Brain Communications* to be at the forefront of this rediscovery. For these two journals to best support progress in clinical and translational neuroscience, we welcome contributions from our colleagues across the fields of psychiatry and behavioural neuroscience as well as our more traditional authors from neurology.
